# Identification of Copy Number Variants as a Suspected Cause of Cerebral Small Vessel Disease

**DOI:** 10.1111/cge.70233

**Published:** 2026-08-27

**Authors:** Solomon K. Guyler, Neven Maksemous, Rodney A. Lea, Robert A. Smith, Heidi G. Sutherland, Lyn R. Griffiths

**Affiliations:** ^1^ Genomics Research Centre, Centre for Genomics and Personalised Health, School of Biomedical Sciences Queensland University of Technology (QUT) Brisbane Queensland Australia; ^2^ Central Analytical Research Facility (CARF) Queensland University of Technology (QUT) Brisbane Queensland Australia

**Keywords:** biology, exome sequencing, genetic testing, genetic variation, genetics, SNP genotyping, vascular dementia

## Abstract

Cerebral small vessel disease (CSVD) describes a range of neurological diseases affecting the small arteries, veins, and capillaries which supply the white matter and deep grey matter structures of the brain. They are the most common form of cerebrovascular disease, accounting for almost half of vascular dementia cases and approximately 20% of stroke incidence globally. Genetic testing is a routine diagnostic tool for monogenic CSVDs; however, less than 20% of patients have a causal variant in a known gene. Genetic testing for these disorders focuses on single nucleotide variants and short insertions or deletions, with larger genomic variation often unexplored as a cause of disease. In this study we performed whole‐exome sequencing (WES) on 111 patients suspected of familial CSVD that had previously tested negative for pathogenic variants in seven known CSVD genes (*NOTCH3*, *HTRA1*, *COL4A1*, *COL4A2*, *TREX1*, *GLA*, and *FOXC1)*. Bioinformatic analysis of WES data, multiplex ligation‐dependent probe amplification, quantitative real‐time polymerase chain reaction assays, and Nanopore long‐read sequencing were used to identify suspected copy number variants. This work identified four candidate CNVs across *NOTCH3*, *LMNB1*, and *COL4A2* which are potential causes of CSVD and highlights the need for further investigation of more complex forms of genetic variation and their potential roles as causal of CSVD.

## Graphical abstract

Whole‐exome sequencing of 111 patients with suspected familial cerebral small vessel disease (CSVD) identified novel copy number variants in four patients across *NOTCH3*, *LMNB1*, and *COL4A2*, using bioinformatic and molecular techniques. These validated CNVs suggest structural variation is an underrecognized potential causal contributor to monogenic CSVD pathogenesis.

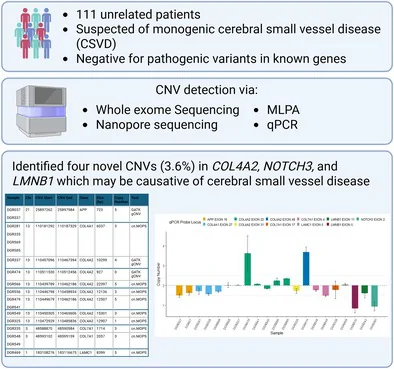

## 1 Introduction

Copy number variation (CNV) refers to structural changes within DNA resulting in altered dosage of genomic segments, which can range in size from several hundred base pairs to 20 megabases (Mb) [1]. CNVs can result from structural variations such as insertions, deletions, and duplications, as well as unbalanced chromosomal rearrangements which may be caused by translocations and inversions [2]. CNVs appear to be widespread throughout the human genome of healthy individuals, and many do not have any association with disease phenotypes [3]. Despite this, CNVs have been implicated in a range of human diseases, including intellectual disability, cancer, and immune deficiency [4]. CNVs have been implicated as causal of several neurological disorders including Charcot–Marie‐Tooth neuropathy and Parkinson's disease, as well as leukoencephalopathy conditions such as autosomal dominant leukodystrophy (ADLD) [5].

Cerebral small vessel disease (CSVD) refers to a group of disorders affecting the brain's small arterial and venous structures. Common manifestations include white matter lesions, lacunes, small infarcts, and subcortical microbleeds, often resulting in cognitive decline and vascular dementia [6]. A substantial proportion of CSVD cases are genetically inherited, with the number of implicated genes causing these disorders continuing to grow [7]. The most frequent monogenic form of CSVD is cerebral autosomal dominant arteriopathy with subcortical infarcts and leukoencephalopathy (CADASIL), caused by cysteine‐altering variants in *NOTCH3* [8]. Other well‐characterised forms of CSVD include: CADASIL2, caused by heterozygous variants in *HTRA1*; Collagen Type 4‐related CSVD, caused by variants in *COL4A1* and *COL4A2*; and Fabry disease, caused by heterozygous variants in *GLA* [9]. While molecular genetic testing of established CSVD genes forms the basis of current diagnostics, such testing only explains ~15% of suspected monogenic CSVD cases, suggesting additional genes or mechanisms remain undiscovered [10].

Genetic diagnosis of CSVD typically involves the investigation of SNVs and short insertion/deletion variants using next generation sequencing (NGS) methods, such as targeted panels or whole‐exome sequencing (WES). However, the detection of larger or more complex structural variation remains challenging from NGS data due to the generation of short read lengths, resulting in an inability to span or map larger variations effectively [11]. Recently, duplications of the *COL4A1* and *COL4A2* genes have been shown to cause CSVD within several families, indicating that CNVs may be an underrepresented cause of inherited CSVD [12, 13]. In this study, we applied two CNV detection algorithms to WES data and performed multiplex ligation‐dependent probe amplification (MLPA) assays to screen for CNVs in genes implicated in monogenic CSVD. Validation of candidate CNVs was performed using quantitative real‐time polymerase chain reaction (qPCR), and long‐read sequencing was utilised to accurately assess CNV size and breakpoints.

## 2 Methods

### 2.1 Overview

CNV analysis was performed in a cohort of 111 patients referred for genetic testing for suspected CADASIL or other monogenic CSVD (Figure 1). CNVs were initially identified from WES data of 111 patients using two independent algorithms, cn.MOPS and the GATK gCNV caller. Additionally, MLPA assays were performed in a subset of 75 samples targeting NOTCH3, PLP1, and LMNB1. All CNVs identified by either bioinformatic or MLPA approaches underwent orthogonal validation using qPCR. Validated CNVs detected originally by MLPA were subsequently characterised using ONT long‐read sequencing, enabling high‐accuracy delineation of CNV breakpoints and size.

**FIGURE 1:**
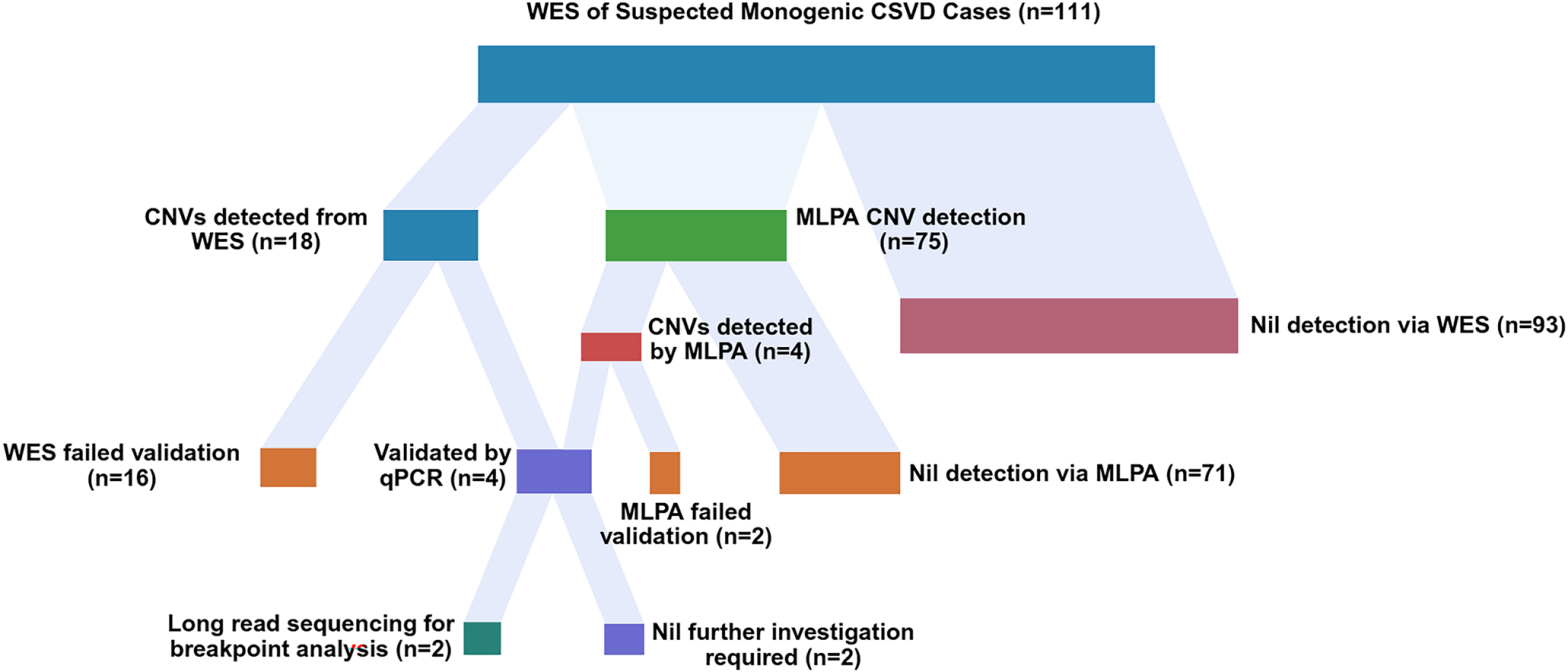
Overview of analysis for suspected monogenic cerebral small vessel disease cases. [Colour figure can be viewed at wileyonlinelibrary.com]

### 2.2 Patient Cohort

One hundred and eleven patients who were referred to the Genomics Research Centre (Queensland University of Technology, Brisbane, Australia) between 1997 and 2025 for molecular genetic testing for monogenic CSVD were included in this study. Participants were referred by neurologists within Australia and New Zealand and selected based upon (a) a clinical suspicion of monogenic CSVD, including magnetic resonance imaging (MRI) findings (white matter hyperintensities, lacunes, microbleeds), subcortical ischemic stroke, family history of cerebral vascular disease, migraine, gait disturbance, cognitive impairment, and vascular dementia; and (b) tested negative for pathogenic variants in seven monogenic CSVD genes, including *NOTCH3*, *HTRA1*, *COL4A1*, *COL4A2*, *TREX1*, *FOXC1*, and *GLA* (Table S1). This study was approved by the Queensland University of Technology (QUT) Ethics Committee, Brisbane, Queensland, Australia (Approval number: 18000007416).

### 2.3 Whole Exome Sequencing

DNA was extracted from peripheral blood samples using the QIAGEN QIACUBE system as per the manufacturer's instructions. Sample quantity was quantified using the Invitrogen Qubit 3 Fluorometer (Thermo Fisher Scientific) using the high‐sensitivity dsDNA quantification kit. WES was performed using Ion AmpliSeq Exome RDY‐kits (Thermo Fisher Scientific) for library preparation as per manufacturers' instructions (MAN0010084). Library quantification was performed using the Qubit 3 Fluorometer for equimolar pooling. Template preparation, enrichment and chip loading were performed using the Ion 550 Kit‐Chef and Ion 550 Chips on the Ion Chef instrumentation. Sequencing was performed using the Ion S5 sequencing platforms (Thermo Fisher Scientific). Raw sequences were aligned to the human genome 38 (hg38) and variant calling was performed using PathVAR, a custom analysis pipeline for NGS data [14]. Coverage and mapping parameters for patient samples showed an average coverage of 118X and mean number of reads of 39 275 917, with an average of 96% of reads on target and an average > 20X coverage of 88.9%.

### 2.4 Control Cohort Generation

The control cohort used in this study was derived from the UK Biobank whole genome sequencing (WGS) resource [15]. A subset of 1035 individuals (mean age = 53, SD = 7.3) with no history of neurological or cerebrovascular disease was selected as a control cohort for this study. WES data for these individuals was extracted from WGS base alignment mapping (BAM) files using the BCFTools package [16].

### 2.5 CNV Calling

Copy number variations were analysed using two bioinformatic tools, cn.MOPS and the GATK gCNV caller [17, 18]. In brief, the cn.MOPS algorithm functions by first mapping reads to fixed‐size windows of non‐overlapping segments in which reads are counted. The model calls CNVs on the assumption that read counts in a segment follow a Poisson distribution, where a mixture model is employed where each observed count is determined across multiple samples to estimate the expected read count per segment, and thus the copy number state for each sample in that segment. The default parameters for cn.MOPS were used for CNV detection, where segments inferred to have a diploid copy number (CN = 2), indicating no copy number change, were excluded from further analysis.

The GATK‐gCNV caller employs a method where coverage information from aligned reads is collected for predefined genomic ranges and coverage outliers and unmappable sequences removed. Samples with similar genomic interval read‐depth profiles are clustered and processed together, and chromosomal ploidies are determined using read‐depth of each chromosome. The algorithm then performs CNV calling using probabilistic modelling. GATK‐gCNV calling was performed using default parameters in cohort‐mode. CNVs with a quality score after calling of < 30 were excluded and additional filtering using a quality state (QS) ≥ 30 was applied to prioritise high‐quality calls. CNVs were compared between cases and controls for both bioinformatic tools, where CNVs with > 1 base pair overlap between both cohorts were removed as likely benign variation. The resulting CNVs calls were then assessed for overlap with known CSVD genes (Table 1).

**TABLE 1:** Cerebral small vessel disease genes and disease associations.

Gene	Disease association (OMIM)
*NOTCH3*	CADASIL (125310)
*HTRA1*	CARASIL/CADASIL2 (600 142/616779)
*COL4A1*	Gould syndrome 1 (175780)
*COL4A2*	Gould syndrome 2 (614483)
*TREX1*	Retinal vasculopathy with cerebral leukoencephalopathy and systemic manifestations (192395)
*FOXC1*	FOXC1‐associated CSVD
*GLA*	Fabry disease (301500)
*ABCC6*	Pseudoxanthoma elasticum (264 800/177850)
*APP*	Cerebral amyloid angiopathy (605714)
*ITM2B*	Familial dementia (176 500/117300)
*ADA2*	Vasculitis, autoinflammation, immunodeficiency, and hematologic effects syndrome (615688)
*CST3*	Cerebral amyloid angiopathy (105150) Autosomal dominant adult‐onset leukodystrophy (621214)
*ARHGEF15*	Cerebral small vessel disease with osteoporosis (621331)
*CSF1R*	Diffuse hereditary leukoencephalopathy with spheroids (221820) Brain abnormalities, neurodegeneration, and dysosteosclerosis (618476)
*LAMB1*	Leukoencephalopathy with hippocampal memory defects (621 424/615191)
*COLGALT1*	Cerebral small vessel disease 3 (618360)
*PLOD3*	Cerebral small vessel disease
*INHA*	Familial CSVD association
*VWA5B2*	Familial CSVD association
*FAM20C*	Familial CSVD association
*LAMC1*	Familial CSVD association

*Note:* Gene transcripts: NOTCH3: NM_000435.3; HTRA1: NM_002775.5; COL4A1: NM_001845.6; COL4A2: NM_001846.4; TREX1: NM_033629.6; FOXC1: NM_001453.3; GLA: NM_000169.3; ABCC6: NM_001171.6; APP: NM_000484.4; ITM2B: NM_021999.5; ADA2: NM_001282225.2; CST3: NM_00099.4; ARHGEF15: NM_173728.4; CSF1R: NM_001288705.3; LAMB1: NM_002291.3; COLGALT1: NM_024656.4; PLOD3: NM_001084.5; INHA: NM_002191.4; VWA5B2: NM_001390846.1; FAM20C: NM_ 001012971.4; LAMC1: NM_002293.4.

### 2.6 MLPA Assay

Genomic DNA was isolated and quantified as previously described prior to assay. MLPA assays were performed using 34 pairs of probes contained in the SALSA MLPA Probemix P071 LMNB1‐PLP1‐NOTCH3 (MRC Holland). The regions targeted by this probe mix include 5q23.2, Xq22.2, and 19p13.12, providing coverage of exons 1–11 of *LMNB1*, exons 2–7 for *PLP1*, and exons 2, 7, and 28 of *NOTCH3*. Assays were performed as per the manufacturer's instructions (Version 010) and fragment separation by capillary electrophoresis was performed using the ABI‐3500 Genetic Analyzer (Thermo Fisher Scientific). The resulting data was analysed using the publicly available analysis software Coffalyser.Net (version 22 040; MRC Holland) using default settings, and comparative analysis was performed using advanced slope correction. Fragment run separation score (FRSS) and fragment MLPA reaction score (FMRS) cutoffs were set at ≥ 75 to ensure sufficient assay quality.

CNVs were detected through examination of ratio charts where duplications and deletions displayed probe ratios which cross the arbitrary borders, and in which there was no overlap between error bars and confidence intervals. Final ratios were extracted from Coffalyser.Net with ≤ 0.7 considered sufficient to indicate heterozygous deletion and ≥ 1.3 sufficient for heterozygous duplication. Samples with final probe ratios of ≤ 0.8 or ≥ 1.2 were retained as suggestive CNV calls.

### 2.7 Quantitative Real‐Time PCR

qPCR was performed to validate suspected CNVs detected by MLPA and WES CNV calling. Primers for qPCR were designed using the NCBI primer design tool with a target amplicon size of 110 base pairs. In addition to the targeted genes, forward and reverse primers were also designed for *GAPDH* for use as a reference gene during analysis (Table S2). Primers were optimised for PCR using concentration and temperature gradients.

Standard curve analysis was performed for optimised primer sets using serial 1 in 2 ratio dilutions, with an initial concentration of 60 ng gDNA and a final dilution concentration of 3.75 ng. Each dilution series was performed in triplicate. Primer sets were considered acceptable when the amplification efficiency was between 90% and 110%. A minimum *R* ^2^ value of 0.99 was used for each primer set to ensure confidence in the assay and that the DNA input was amplifying consistently across multiple concentrations.

Following confirmation of primer optimization and specificity, qPCR assays were performed for each patient sample with a suspected CNV using the Promega GoTaq qPCR kit as per the manufacturer's instructions (#FB103) at a one‐half final reaction volume (10 μL). Assays were performed using the QuantStudio Real Time PCR System (Applied Biosystems). Analysis of qPCR data was performed using relative quantification where the 2^−ΔΔCT^ method was used to calculate copy number change, based on the assumption that the control sample is diploid for each of the target genes. CNV calls were considered positive if fold change was < 1.5 for heterozygous deletions and > 2.5 for heterozygous duplications. qPCR assays were performed in triplicate across three independent runs to ensure assay reproducibility.

### 2.8 ONT Adaptive Sampling

Oxford Nanopore Technologies (ONT) adaptive sampling was performed for samples with validated CNVs detected by MLPA due to incomplete coverage of exons within target genes. Sequencing libraries were prepared from 1 μg of input DNA using Ligation Sequencing Kits (SQK‐LSK110; Oxford Nanopore Technologies) and R10.4.1 MinION flow cells prepared according to the manufacturer's instructions (V GDE_9161_v114_revAC_24Sep2025). When the purified library concentration exceeded 300 ng, final libraries were diluted to 300 ng in a total volume of 12 μL for sample loading; otherwise, the entire library was loaded. Sequencing was performed on a MinION Mk1B device for a total runtime of 30–35 h with flow cells washed and reloaded with sequencing libraries after 20–24 h of runtime. Adaptive sampling was configured using a custom BED file containing the chromosomal coordinates of 81 genes associated with CSVD, migraine, or related neurological disorders (Table S3). Genes were padded with 500 kb upstream and downstream to ensure the region of interest (ROI) totalled at least 2% of the genome, for a total of 90 440 933 bp of target sequence, which was uploaded to MinKNOW v25.05.14.

Base calling was performed using dorado (v1.2.0) with a minimum quality score (*Q* score) of 8. Sequencing reads were mapped using minimap2 (v2.30‐r1287) to the reference genome hg38. Mapped reads were then input into the wf‐human‐variation workflow (v2.7.3) and CNVs assessed using Sniffles (v2.7.4) and visual inspection of breakpoints in the Integrated Genomics Viewer (IGV) (v2.17.0).

### 2.9 In Silico Prediction of Identified Variants

To evaluate the clinical significance of candidate CNVs, three in silico prediction tools were applied. ClassifyCNV classifies CNVs according to 2019 ACMG/ClinGen guidelines, while CNVoyant is a machine‐learning classification algorithm trained on ClinVar‐annotated CNVs, and X‐CNV generates a meta‐voting prediction (MVP) score indicating pathogenicity using a machine‐learning model trained on genomic and population‐frequency features [19, 20, 21]. Each tool was applied to candidate CNVs using GRCh38 coordinates.

## 3 Results

CNV calling from WES data was performed for a cohort of 111 CSVD patients using two bioinformatic tools, cn.MOPS and the GATK gCNV caller (gCNV). gCNV identified 395 total suspected CNVs while cn.MOPS identified 6226 total CNVs. Analysis of CSVD genes identified 12 unique CNVs across 18 patients (*n* = 18/111; 16.2%), including four CNVs detected by gCNV and eight CNVs detected by cn.MOPS (Table 2). The majority of CNVs were detected in *COL4A2* (*n* = 7). A 6037 bp duplication encompassing exons 25–29 of *COL4A1* was observed in four patients. Additionally, two patients carried an identical 2057 bp duplication in *COL7A1*, and two others exhibited a five‐fold amplification spanning 723 bp of *APP*, encompassing exon 16.

**TABLE 2:** Copy number variations detected by WES analysis.

Sample	Chr	CNV Start	CNV End	Gene	Size (bp)	Copy Number	Tool
DGR037	21	25897262	25897984	*APP*	723	5	GATK gCNV
DGR337	21	25897262	25897984	*APP*	723	5	GATK gCNV
DGR281	13	110181292	110187329	*COL4A1*	6037	3	cn.MOPS
DGR335	13	110181292	110187329	*COL4A1*	6037	3	cn.MOPS
DGR569	13	110181292	110187329	*COL4A1*	6037	3	cn.MOPS
DGR585	13	110181292	110187329	*COL4A1*	6037	3	cn.MOPS
DGR337	13	110457096	110467394	*COL4A2*	10 299	4	GATK gCNV
DGR474	13	110511530	110512456	*COL4A2*	927	0	GATK gCNV
DGR566	13	110439789	110462186	*COL4A2*	22 397	5	cn.MOPS
DGR556	13	110446798	110458934	*COL4A2*	12 136	3	cn.MOPS
DGR479	13	110449679	110462186	*COL4A2*	12 507	5	cn.MOPS
DGR541	13	110449769	110462186	*COL4A2*	12 507	5	cn.MOPS
DGR549	13	110450305	110465606	*COL4A2*	15 301	3	cn.MOPS
DGR325	13	110472929	110485836	*COL4A2*	12 907	1	cn.MOPS
DGR335	3	48588870	48590584	*COL7A1*	1714	3	cn.MOPS
DGR548	3	48593102	48595159	*COL7A1*	2057	3	cn.MOPS
DGR549	3	48593102	48595159	*COL7A1*	2057	3	cn.MOPS
DGR469	1	183108276	183116675	*LAMC1*	8399	5	cn.MOPS

*Note:* Gene transcripts: *APP*: *NM_000484*.*4*; *COL4A1*: *NM_001845*.*6*; *COL4A2*: *NM_001846*.*4*; *COL7A1*: *NM_000094*.*4*; *LAMC1*: *NM_002293*.*4*.

MLPA assays were performed for 75 patient samples to assess CNVs across *NOTCH3*, *LMNB1*, and *PLP1*. Three suspected CNVs were identified across three patients, including one heterozygous deletion affecting exon two of *NOTCH3* in DGR350, and one heterozygous deletion affecting exon five of *LMNB1* in DGR570 (Table 3). One potential heterozygous duplication was identified in *LMNB1* exon 11 with a final ratio of 1.28 and was subsequently retained as a potential duplication. No suspected CNVs were identified in *PLP1*.

**TABLE 3:** Candidate mutations identified through MLPA assay.

Sample	Gene	Exon	Final ratio	Standard deviation
DGR350	*NOTCH3*	2	0.69	0.04
DGR570	*LMNB1*	5	0.67	0.05
DGR342	*LMNB1*	11	1.28	0.05

Validation of suspected CNVs detected by MLPA or bioinformatic analysis of WES data was performed using qPCR. Four suspected CNVs were confirmed by qPCR, including both heterozygous deletions detected by MLPA, as well as two duplications in DGR474 and DGR479 in *COL4A2* which were initially detected via copy number caller analysis of WES data (Figure 2). The amplification of exons 19–23 of *COL4A2* in DGR479 was detected as a 5‐fold copy number gain by cn.MOPS while qPCR identified an average copy number fold increase of 3.6 copies. The CNV in exon 48 of *COL4A2* in DGR474 was initially detected as a homozygous deletion by GATK gCNV; however, qPCR detected an average copy number fold increase of 3.7 copies indicating amplification of this locus. The remaining suspected CNV calls identified from WES data or MLPA failed to replicate by qPCR, representing a positive validation rate from WES data of 33.3% (*n* = 4/12).

**FIGURE 2:**
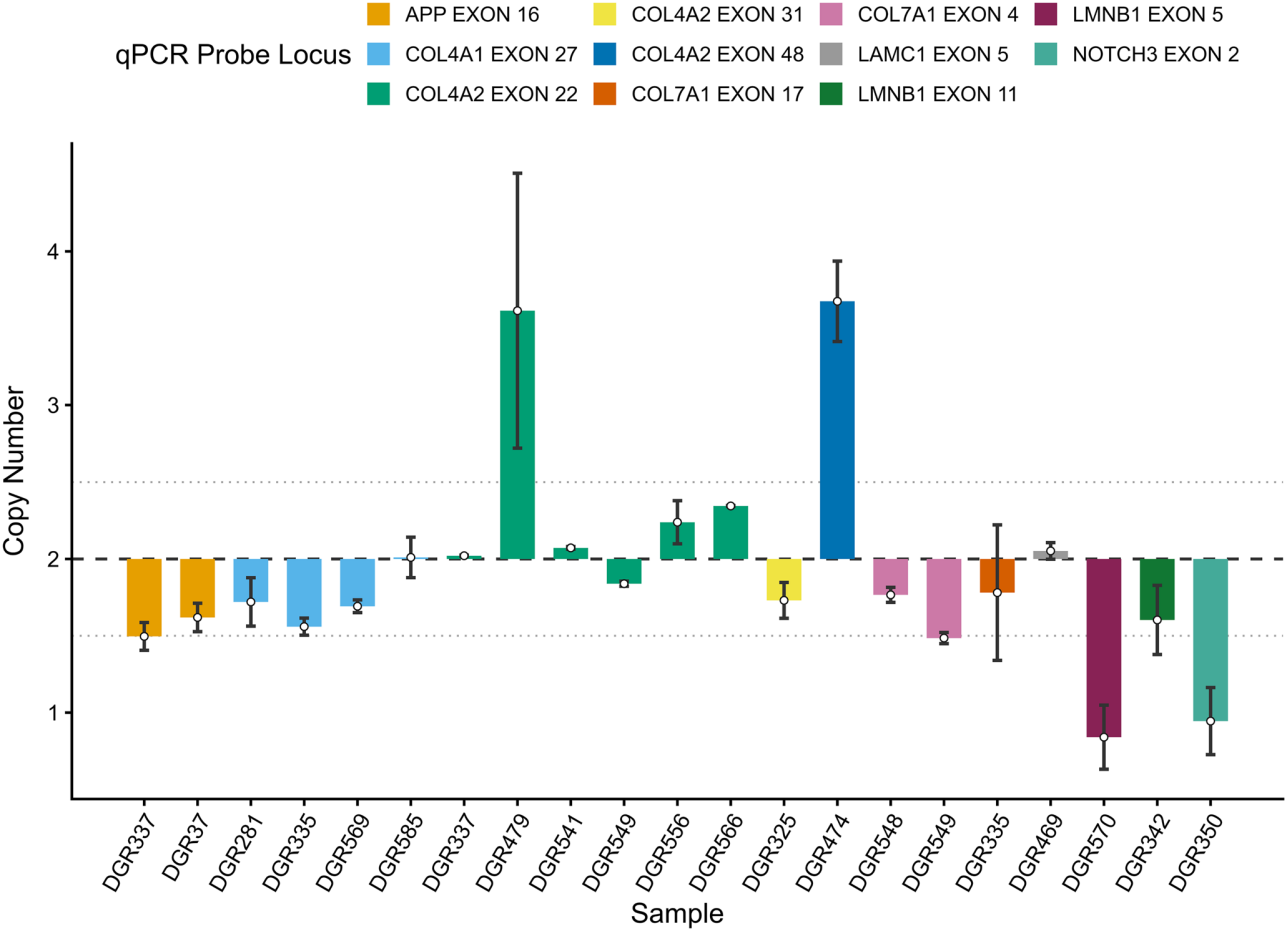
qPCR relative fold change for suspected copy number variants. [Colour figure can be viewed at wileyonlinelibrary.com]

Long‐read sequencing was performed for both heterozygous deletions identified from MLPA using ONT adaptive sampling. Nanopore sequencing identified both heterozygous deletions, supported by a ~50% reduction in coverage spanning the 5′ UTR and exons one and two of *NOTCH3* in DGR350, and exon 5 of *LMNB1*, as well as multiple split‐reads at both junctions (Table 4). Breakpoints were determined to fall several hundred bases upstream and downstream for both heterozygous deletions.

**TABLE 4:** Copy number variants identified by nanopore adaptive sampling.

Sample	Locus	Size (bp)	Copy number
DGR570	chr5:126811157‐126811955	798	1
DGR350	chr19:15197483‐15202155	4672	1

*Note:* All genomic coordinates are based on the GRCh38 (hg38) human genome assembly.

Pathogenicity prediction for identified CNVs was performed using ClassifyCNV, X‐CNV, and CNVoyant (Table 5). ClassifyCNV classified all four variants as of uncertain significance. X‐CNV and CNVoyant classified the *NOTCH3* deletion in DGR350 as likely pathogenic and pathogenic respectively, while the *LMNB1* deletion in DGR570 was classified as pathogenic by both tools. For the two *COL4A2* duplications, the DGR479 variant spanning exons 19–23 was classified as likely benign by X‐CNV and benign by CNVoyant, while the DGR474 variant affecting exon 48 was classified as uncertain by X‐CNV and benign by CNVoyant.

**TABLE 5:** In silico classification of candidate CNVs.

Variant	CNV Type	X‐CNV (MVP Score)	CNVoyant	ClassifyCNV
chr13:110511530‐110512456	Duplication	Uncertain (0.450)	Benign	Uncertain significance (0)
chr13:110449679‐110462186	Duplication	Likely Benign (0.154)	Benign	Uncertain significance (0)
chr5:126811157‐126811955	Deletion	Pathogenic (0.854)	Pathogenic	Uncertain significance (0)
chr19:15197483‐15202155	Deletion	Likely Pathogenic (0.755)	Pathogenic	Uncertain significance (0)

*Note:* X‐CNV classifications are reported alongside their corresponding meta‐voting prediction (MVP) score (range 0–1). ClassifyCNV classifications are reported alongside their ACMG/ClinGen evidence score. All variants were assessed using GRCh38 coordinates.

## 4 Discussion

In this study, we identified four true positive CNVs across three genes causative of leukoencephalopathies, representing a 3.6% variant detection rate in this suspected CSVD cohort. Two CNVs in DGR350 and DGR570 were initially detected using MLPA and were subsequently validated using qPCR and long‐read sequencing via ONT adaptive sampling, while an additional 12 CNVs were detected from WES data, of which two were confirmed by qPCR in DGR474 and DGR479. Both CNVs identified from WES data were detected by cn.MOPS, while no CNVs detected by the GATK gCNV caller were able to be validated using additional methods.

### 4.1 *NOTCH3* Variant

One heterozygous deletion was detected in *NOTCH3* in DGR350, which was shown to result in the deletion of exons one and two containing both the 5′ UTR and the canonical start codon. CADASIL is typically caused by heterozygous missense variants which alter cysteine residues within the EGFr regions of *NOTCH3*, located in exons 2–24. These variants disrupt disulphide bridge formation and lead to aberrant folding and alter the tertiary structure of the mature NOTCH3 protein, ultimately resulting in a toxic gain‐of‐function [22]. The heterozygous deletion observed here is likely to result in the loss of the canonical start codon and thus result in the loss of the functional protein product due to nonsense‐mediated decay or protein truncation. Homozygous loss‐of‐function (LoF) variants in *NOTCH3* are causative of an early‐onset leukoencephalopathy and prominent neurodevelopmental features while heterozygous LoF variants have been associated with a CSVD which appears distinct from CADASIL in disease severity but may be exacerbated by cardiovascular risk factors and age [8, 23].

The association between protein‐truncating variants and CADASIL remains uncertain, with null variants resulting in reduced NOTCH3 signalling and expression but lacking the pathognomonic granular osmiophilic material (GOM) [24, 25]. DGR350, a 60‐year‐old female, presented with a history of increased confusion and cognitive decline over several years, with white matter changes typical of CADASIL on MRI. The patient had additionally been diagnosed with vascular dementia and had a history of seizures. Notably, an identical twin sister was also reported to be affected; however, she was unavailable for familial segregation and, as such, zygosity is unknown. The strong CSVD phenotype observed here and the variant prediction as pathogenic/likely pathogenic by X‐CNV and CNVoyant indicates this variant is likely to be causative of disease in this patient.

### 4.2 *LMNB1* Variant

A 798 bp deletion was identified in DGR570 involving the *LMNB1* gene, which is implicated in autosomal dominant leukodystrophy (ADLD). ADLD results from whole‐gene duplications of *LMNB1* or more rarely upstream regulatory deletions resulting in *LMNB1* overexpression [26]. The heterozygous deletion identified here results in the loss of exon five and is likely to result in alternate splicing or the production of a truncated protein due to preservation of the reading frame [27]. Further, the interactions between the lamin B1 coiled‐coil domain of the dimer are essential to tetramerization, where the interaction of α‐helical structures of each dimer promotes antiparallel interactions between them, resulting in the longitudinal assembly of lamins in the nuclear lamina [28]. The loss of 42 amino acids from this domain is therefore likely to be deleterious to protein function and affect protein abundance in the nucleus, impairing nuclear envelope stability [29].

While this variant may not result in overexpression of *LMNB1* due to gene duplication, disruption of the α‐helical rod domain is likely to result in altered or impaired protein function. Reductions in *LMNB1* expression have been associated with neurodegenerative disease including Alzheimer's disease and Parkinson's disease due to its role in neurogenesis [30]. DGR570, a 41‐year‐old male, presented with a history of leukoencephalopathy and migraine, consistent with the white matter disease phenotype associated with *LMNB1*‐related disorders, and the deleteriousness of this variant is supported by X‐CNV and CNVoyant which classified the variant as pathogenic [31].

### 4.3 *COL4A2* Variants

Two copy number amplifications within *COL4A2* were identified in DGR474 and DGR479. *COL4A2* encodes the α2 chain of Type IV collagen, which assembles with two α1 chains encoded by *COL4A1* in the endoplasmic reticulum to form a heterotrimer. The mature heterotrimer is subsequently secreted into the extracellular matrix, where it forms a core structural component of the vascular basement membrane. Pathogenic *COL4A2* variants are a well‐established cause of familial porencephaly, intracerebral hemorrhage, and white matter disease [32]. Structural variation in *COL4A2* is rare; however, a growing body of evidence has linked duplications of *COL4A1* and *COL4A2* with overexpression of the mature protein and a resulting CSVD phenotype [12, 13, 33, 34].

The copy number amplification observed in DGR479 encompasses exons 19–23 of *COL4A2* and is likely to cause a frameshift as exon 19 begins with a phase one intron, resulting in the introduction of a premature stop codon downstream and resulting in nonsense‐mediated decay of the resultant protein product. Nonsense variants resulting in LoF have been shown to cause *COL4A2*‐related CSVD and result in abnormalities of the basement membrane of small blood vessels, intracellular accumulation of protein products, and increased apoptosis [35]. DGR479, a 48‐year‐old female, presented with a history of migraine and white matter hyperintensities on MRI, consistent with the features observed in *COL4A2*‐related CSVD. In silico classification of this variant was variable, with X‐CNV and CNVoyant classifying it as likely benign and benign respectively, while ClassifyCNV returned a classification of uncertain significance. As such, the interpretation of this variant is unclear yet may be causative of disease in this patient.

DGR474, a 53‐year‐old female, presented with a history of recurrent subcortical ischemic infarcts. The copy number amplification identified in this patient affects exon 48, the last exon in *COL4A2*, which encodes the non‐collagenous domain at the C‐terminus [36]. This region is essential to the initiation of formation of heterotrimers [37], and the incorporation of additional copies of this region in tandem is likely to destabilise trimerization and interactions with other proteins in the ECM. Similarly to DGR479, *in silico* prediction of this variant was discordant with the majority of tools identifying it as uncertain significance, and its role as a cause of disease remains unclear.

The four confirmed CNVs identified in this study represent compelling candidates as contributors to CSVD pathogenesis. The accuracy of CNV detection is inherently constrained by the limitations of available methodologies. This is particularly evident in CNVs inferred from short‐read WES data, where variability in capture efficiency across exonic regions and fluctuations in read depth can generate artifactual CNV signals that are difficult to distinguish from true variants without orthogonal validation. Accordingly, although 12 CNVs were detected bioinformatically, only a subset could be orthogonally confirmed using laboratory‐based methods. While both the GATK gCNV caller and cn.MOPS are robust tools for CNV detection from WES data and demonstrate high accuracy in benchmark datasets, sensitivity and precision vary significantly between calling algorithms and datasets [38]. CNV detection is inherently more robust when performed using whole‐genome sequencing (WGS), which provides more uniform coverage and improved breakpoint resolution, and may enable more accurate detection of CNVs within this cohort [17].

The MLPA probe sets used within this study only provide coverage of three exons of *NOTCH3*, and additional CNVs may be present in unexamined regions within this cohort. Further studies involving analysis of gene expression and protein function and stability are essential to determining biological consequences of the identified variants and enabling robust pathogenicity classification. The retrospective nature of this cohort, recruited across several decades and through multiple referring clinicians, precluded access to standardised clinical and neuroimaging data, limiting the ability to draw robust genotype–phenotype correlations. Familial DNA was also unavailable for the majority of patients, preventing segregation, penetrance, and de novo analyses, and as such we cannot exclude that identified variants occurred *de novo*.

CNVs are routinely understudied as potential causes of inherited CSVD. Consequently, classification of pathogenicity using relevant guidelines, such as those published by the American College of Medical Genetics (ACMG), may be inaccurately applied due to the rarity of precedent cases and limited functional data [39, 40]. In such cases, familial segregation analysis, longitudinal clinical follow‐up, functional characterisation, and literature reporting or submission to variant databases are crucial to facilitate evidence accumulation and support future reclassification of candidate variants.

## 5 Conclusion

This study identified four potential causal CNVs across four patients suspected of monogenic CSVD in a cohort of 111 patients in which no pathogenic single nucleotide variants were detected. None of the variants identified here has previously been detected and may contribute to disease in this cohort. Collectively, these findings highlight CNVs as an important yet under‐detected class of variation contributing to hereditary cerebral small vessel disease. Incorporating CNV screening techniques into diagnostic pipelines may detect structural variants that contribute to disease in suspected monogenic CSVD and improve the efficacy of diagnostic testing.

## Funding

This work was supported by the Australian National Health and Medical Research Council (NHMRC‐APP1122387) (LRG), by infrastructure purchased with Australian Government EIF Super Science Funds as part of the Therapeutic Innovation Australia—Queensland Node project (LRG), and by a Queensland University of Technology Postgraduate Research Award (QUTPRA).

## Ethics Statement

The study was approved by the Human Research Ethics Committee of the Queensland University of Technology (approval number: 14000007416; approval date: June 12, 2023) with appropriate written consent obtained from patients and in accordance with the Declaration of Helsinki. Diagnostic genetic testing was conducted under National Association of Testing Authorities (NATA) accreditation (accreditation number 14979).

## Conflicts of Interest

The authors declare no conflicts of interest.

## Supporting information


**Table S1:** Clinical and Demographic Features of Case and Control Cohorts.
**Table S2:** qPCR primer design of amplicon sequences.
**Table S3:** Chromosomal coordinates of genes included in BED file for Oxford Nanopore long‐read sequencing.

## Data Availability

The data that support the findings of this study are available on request from the corresponding author. The data are not publicly available due to privacy or ethical restrictions.
