# Supplementary material for: Identification of Copy Number Variants as a Suspected Cause of Cerebral Small Vessel Disease
Source: Clin Genet. 2026 Aug 27;110(6):686–94. doi: 10.1111/cge.70233 (PMC13728972; doi:10.1111/cge.70233)
Supplement: Supplementary file 1 [file CGE-110-686-s001.docx]

|  | Case (%) | Control (%) |
| --- | --- | --- |
| **Demographics** | | |
| *n* | 111 | 1035 |
| Age at referral | 52.3 ± 12.7 | 53 ± 7.3 |
| Sex, male | 35 (31.5) | 518 (50) |
| **CSVD MRI Markers** | | |
| WMH | 90 (81.1) | N/A |
| Lacunes | 17 (15.3) | N/A |
| CMB | 15 (13.5) | N/A |
| **Clinical Features** | | |
| Ischaemic stroke | 33 (29.7) | 0 |
| ICH | 1 (0.9) | 0 |
| Migraine | 31 (27.9) | 0 |
| Vascular dementia | 5 (4.5) | 0 |
| Ocular abnormalities | 3 (2.7) | 0 |
| Cognitive decline | 14 (12.6) | 0 |
| Psychiatric disturbance | 5 (4.5) | 0 |
| Gait disturbance | 9 (8.1) | 0 |
| Cerebral vascular disease | 5 (4.5) | 0 |
| Family history of cerebral vascular disease | 13 (11.7) | N/A |
| Skin biopsy changes | 6 (5.4) | 0 |
| **Vascular Risk Factors** | | |
| Hypertension | 0 | N/A |
| Hyperlipidaemia | 0 | N/A |

*Supplementary Table 1: Clinical and Demographic Features of Case and Control Cohorts*

*Data is presented as n (%) or mean ± standard deviation. Clinical and neuroimaging data were not collected for UK Biobank control participants, who were selected on the basis of confirmed absence of neurological disease or symptoms by questionnaire or interview; these parameters are therefore indicated as not assessed. CSVD, cerebral small vessel disease; WMH, white matter hyperintensities; CMB, cerebral microbleeds; ICH, intracerebral haemorrhage; N/A, not assessed.*

Supplementary Table 2: qPCR primer design of amplicon sequences.

| Gene | Forward | Reverse | Size (bp) |
| --- | --- | --- | --- |
| COL4A1 Exon 27 | ATTGTTCCTTTACCAGGCCCC | ACTCTCGTGGTATCCCGGTG | 110 |
| COL4A2 Exon 21 | CCCTGCATCTGTGGTTGTCT | ACCTTTCCACCCCTTTGGTC | 121 |
| COL4A2 Exon 22 | AATGCGGAACAAGGAGGC | CAGTCCCGGAAGACCTTTGAT | 101 |
| COL4A2 Exon 31 | TCCACCCTGTTTGATTTGCT | GAGAGACCTTTCATGCCCACA | 104 |
| COL4A2 Exon 48 | TGTGATTCCTAACCCTGTCCTG | GTCCTCTAGACAGCTGCCCG | 105 |
| COL7A1 Exon 17 | CAGAGTTTGCTAGCCCTGGA | GTATCTGGCTCCAGTCCATCC | 113 |
| COL7A1 Exon 4 | GTGATTTGACCCCTGCACCT | AGCAAATAGCTTGACCCCCT | 117 |
| LAMC1 Exon 5 | ACCCAGATCTGATGTAACTCGTG | TGGCCAGTGGAACGATAGAG | 105 |
| NOTCH1 Exon 11 | GACCGCGACAACGCCTA | GTGGCACGTGTCGGTCAGT | 120 |
| APP Exon 16 | ACTAATTGGTTGTCCTGCATACTTT | GTCGGAATTCTGCATCCATCT | 109 |
| NOTCH3 Exon 2 | CTGGGCACCTGGCTGATCCT | TGGGTGCAACGACCTCCATTT | 96 |
| LMNB1 Exon 5 | AGACTGACTATGCTTTGCTTCTTCT | TCATGCGGCTTTCCATCAGT | 112 |
| LMNB1 Exon 11 | CATCCAATAGAAGCTGTGCAATTA | GAGAAGGCTCTGCACTGTAT | 100 |
| GAPDH | GGAAACCAGATCTCCCACCG | GTGTGTGGGGAGAAGGGATG | 110 |

*Gene transcripts: APP: NM_000484.4; COL4A1: NM_001845.6; COL4A2: NM_001846.4; COL7A1: NM_000094.4; LAMC1: NM_002293.4; NOTCH1: NM_004557.4; APP: NM_000484.4; NOTCH3: NM_000435.3; LMNB1: NM_005573.4; GAPDH: NM_002046.7.*

*Supplementary Table 3: Chromosomal coordinates of genes included in BED file for Oxford Nanopore long-read sequencing*

| Chromosome | Start | End | Gene | Strand |
| --- | --- | --- | --- | --- |
| chr1 | 2569202 | 3438621 | PRDM16_NM_022114.4_ENST00000270722.10 | + |
| chr1 | 3069202 | 3938621 | PRDM16_NM_022114.4_ENST00000270722.10 | - |
| chr1 | 148890620 | 149471833 | NOTCH2NLC_NM_001364013.2_ENST00000650865.2 | + |
| chr1 | 149390620 | 149971833 | NOTCH2NLC_NM_001364013.2_ENST00000650865.2 | - |
| chr1 | 150049407 | 150560933 | ADAMTSL4_NM_019032.6_ENST00000271643.9 | + |
| chr1 | 150549407 | 151060933 | ADAMTSL4_NM_019032.6_ENST00000271643.9 | - |
| chr1 | 155694103 | 156212796 | SLC25A44_NM_014655.4_ENST00000359511.5 | + |
| chr1 | 156194103 | 156712796 | SLC25A44_NM_014655.4_ENST00000359511.5 | - |
| chr1 | 159615758 | 160143591 | ATP1A2_NM_000702.4_ENST00000361216.8 | + |
| chr1 | 160115758 | 160643591 | ATP1A2_NM_000702.4_ENST00000361216.8 | - |
| chr1 | 159651602 | 160186980 | ATP1A4_NM_144699.4_ENST00000368081.9 | + |
| chr1 | 160151602 | 160686980 | ATP1A4_NM_144699.4_ENST00000368081.9 | - |
| chr1 | 180983516 | 181808084 | CACNA1E_NM_001205293.3_ENST00000367573.7 | + |
| chr1 | 181483516 | 182308084 | CACNA1E_NM_001205293.3_ENST00000367573.7 | - |
| chr1 | 185234390 | 186190949 | HMCN1_NM_031935.3_ENST00000271588.9 | + |
| chr1 | 185734390 | 186690949 | HMCN1_NM_031935.3_ENST00000271588.9 | - |
| chr1 | 214582736 | 215237090 | KCNK2_NM_001017425.3_ENST00000444842.7 | + |
| chr1 | 215082736 | 215737090 | KCNK2_NM_001017425.3_ENST00000444842.7 | - |
| chr1 | 226370615 | 226896098 | PSEN2_NM_000447.3_ENST00000366783.8 | + |
| chr1 | 226870615 | 227396098 | PSEN2_NM_000447.3_ENST00000366783.8 | - |
| chr2 | 55365966 | 55923782 | EFEMP1_NM_001039348.3_ENST00000355426.8 | + |
| chr2 | 55865966 | 56423782 | EFEMP1_NM_001039348.3_ENST00000355426.8 | - |
| chr2 | 164587525 | 165204050 | SCN3A_NM_006922.4_ENST00000283254.12 | + |
| chr2 | 165087525 | 165704050 | SCN3A_NM_006922.4_ENST00000283254.12 | - |
| chr2 | 164739413 | 165392304 | SCN2A_NM_001371246.1_ENST00000631182.3 | + |
| chr2 | 165239413 | 165892304 | SCN2A_NM_001371246.1_ENST00000631182.3 | - |
| chr2 | 165485812 | 166128020 | SCN1A_NM_001165963.4_ENST00000674923.1 | + |
| chr2 | 165985812 | 166628020 | SCN1A_NM_001165963.4_ENST00000674923.1 | - |
| chr2 | 165695184 | 166375944 | SCN9A_NM_001365536.1_ENST00000642356.2 | + |
| chr2 | 166195184 | 166875944 | SCN9A_NM_001365536.1_ENST00000642356.2 | - |
| chr2 | 165903572 | 166494249 | SCN7A_NM_002976.4_ENST00000643258.1 | + |
| chr2 | 166403572 | 166994249 | SCN7A_NM_002976.4_ENST00000643258.1 | - |
| chr2 | 178025988 | 178807423 | TTN_NM_001267550.2_ENST00000589042.5 | + |
| chr2 | 178525988 | 179307423 | TTN_NM_001267550.2_ENST00000589042.5 | - |
| chr2 | 188474372 | 189012746 | COL3A1_NM_000090.4_ENST00000304636.9 | + |
| chr2 | 188974372 | 189512746 | COL3A1_NM_000090.4_ENST00000304636.9 | - |
| chr3 | 38048061 | 38649687 | SCN5A_NM_000335.5_ENST00000423572.7 | + |
| chr3 | 38548061 | 39149687 | SCN5A_NM_000335.5_ENST00000423572.7 | - |
| chr3 | 38196806 | 38816217 | SCN10A_NM_006514.4_ENST00000449082.3 | + |
| chr3 | 38696806 | 39316217 | SCN10A_NM_006514.4_ENST00000449082.3 | - |
| chr3 | 38345763 | 39051944 | SCN11A_NM_001349253.2_ENST00000302328.9 | + |
| chr3 | 38845763 | 39551944 | SCN11A_NM_001349253.2_ENST00000302328.9 | - |
| chr3 | 40746598 | 41962103 | ULK4_NM_017886.4_ENST00000301831.9 | + |
| chr3 | 41246598 | 42462103 | ULK4_NM_017886.4_ENST00000301831.9 | - |
| chr3 | 47966229 | 48467645 | TREX1_NM_033629.6_ENST00000625293.3 | + |
| chr3 | 48466229 | 48967645 | TREX1_NM_033629.6_ENST00000625293.3 | - |
| chr3 | 52994610 | 53813733 | CACNA1D_NM_001128840.3_ENST00000350061.11 | + |
| chr3 | 53494610 | 54313733 | CACNA1D_NM_001128840.3_ENST00000350061.11 | - |
| chr3 | 182293503 | 182921629 | ATP11B_NM_014616.3_ENST00000323116.10 | + |
| chr3 | 182793503 | 183421629 | ATP11B_NM_014616.3_ENST00000323116.10 | - |
| chr5 | 82971743 | 83582302 | VCAN_NM_004385.5_ENST00000265077.8 | + |
| chr5 | 83471743 | 84082302 | VCAN_NM_004385.5_ENST00000265077.8 | - |
| chr5 | 121563194 | 122078259 | LOX_NM_002317.7_ENST00000231004.5 | + |
| chr5 | 122063194 | 122578259 | LOX_NM_002317.7_ENST00000231004.5 | - |
| chr5 | 126277135 | 126837020 | LMNB1_NM_005573.4_ENST00000261366.10 | + |
| chr5 | 126777135 | 127337020 | LMNB1_NM_005573.4_ENST00000261366.10 | - |
| chr5 | 127757908 | 128538245 | FBN2_NM_001999.4_ENST00000262464.9 | + |
| chr5 | 128257908 | 129038245 | FBN2_NM_001999.4_ENST00000262464.9 | - |
| chr5 | 139832842 | 140346603 | HBEGF_NM_001945.3_ENST00000230990.7 | + |
| chr5 | 140332842 | 140846603 | HBEGF_NM_001945.3_ENST00000230990.7 | - |
| chr5 | 149553294 | 150086554 | CSF1R_NM_001288705.3_ENST00000675795.1 | + |
| chr5 | 150053294 | 150586554 | CSF1R_NM_001288705.3_ENST00000675795.1 | - |
| chr6 | 889575 | 1395603 | FOXF2_NM_001452.2_ENST00000645481.2 | + |
| chr6 | 1389575 | 1895603 | FOXF2_NM_001452.2_ENST00000645481.2 | - |
| chr6 | 1109914 | 1613897 | FOXC1_NM_001453.3_ENST00000645831.2 | + |
| chr6 | 1609914 | 2113897 | FOXC1_NM_001453.3_ENST00000645831.2 | - |
| chr6 | 141802006 | 142446261 | ADGRG6_NM_198569.3_ENST00000367609.8 | + |
| chr6 | 142302006 | 142946261 | ADGRG6_NM_198569.3_ENST00000367609.8 | - |
| chr7 | 100705983 | 101217581 | PLOD3_NM_001084.5_ENST00000223127.8 | + |
| chr7 | 101205983 | 101717581 | PLOD3_NM_001084.5_ENST00000223127.8 | - |
| chr7 | 107423798 | 108003161 | LAMB1_NM_002291.3_ENST00000222399.11 | + |
| chr7 | 107923798 | 108503161 | LAMB1_NM_002291.3_ENST00000222399.11 | - |
| chr9 | 70029059 | 71121621 | TRPM3_NM_001366145.2_ENST00000677713.2 | + |
| chr9 | 70529059 | 71621621 | TRPM3_NM_001366145.2_ENST00000677713.2 | - |
| chr9 | 74222494 | 74887921 | TRPM6_NM_017662.5_ENST00000360774.6 | + |
| chr9 | 74722494 | 75387921 | TRPM6_NM_017662.5_ENST00000360774.6 | - |
| chr9 | 114519574 | 115118157 | TNC_NM_002160.4_ENST00000350763.9 | + |
| chr9 | 115019574 | 115618157 | TNC_NM_002160.4_ENST00000350763.9 | - |
| chr9 | 135994432 | 136546048 | NOTCH1_NM_017617.5_ENST00000651671.1 | + |
| chr9 | 136494432 | 137046048 | NOTCH1_NM_017617.5_ENST00000651671.1 | - |
| chr9 | 137377781 | 138124619 | CACNA1B_NM_000718.4_ENST00000371372.6 | + |
| chr9 | 137877781 | 138624619 | CACNA1B_NM_000718.4_ENST00000371372.6 | - |
| chr10 | 103094026 | 103855576 | SH3PXD2A_NM_001394015.1_ENST00000369774.9 | + |
| chr10 | 103594026 | 104355576 | SH3PXD2A_NM_001394015.1_ENST00000369774.9 | - |
| chr10 | 116697488 | 117210299 | KCNK18_NM_181840.1_ENST00000334549.1 | + |
| chr10 | 117197488 | 117710299 | KCNK18_NM_181840.1_ENST00000334549.1 | - |
| chr10 | 121961552 | 122514907 | HTRA1_NM_002775.5_ENST00000368984.8 | + |
| chr10 | 122461552 | 123014907 | HTRA1_NM_002775.5_ENST00000368984.8 | - |
| chr11 | 1904514 | 2444514 | TRPM5_NM_014555.4_ENST00000696290.1 | + |
| chr11 | 2404514 | 2944514 | TRPM5_NM_014555.4_ENST00000696290.1 | - |
| chr11 | 46908589 | 47416496 | SLC39A13_NM_001128225.3_ENST00000362021.9 | + |
| chr11 | 47408589 | 47916496 | SLC39A13_NM_001128225.3_ENST00000362021.9 | - |
| chr11 | 78152828 | 79441030 | TENM4_NM_001098816.3_ENST00000278550.12 | + |
| chr11 | 78652828 | 79941030 | TENM4_NM_001098816.3_ENST00000278550.12 | - |
| chr12 | 1552986 | 2697950 | CACNA1C_NM_000719.7_ENST00000399655.6 | + |
| chr12 | 2052986 | 3197950 | CACNA1C_NM_000719.7_ENST00000399655.6 | - |
| chr12 | 4409904 | 4918256 | KCNA1_NM_000217.3_ENST00000382545.5 | + |
| chr12 | 4909904 | 5418256 | KCNA1_NM_000217.3_ENST00000382545.5 | - |
| chr12 | 51091232 | 51812864 | SCN8A_NM_001330260.2_ENST00000627620.5 | + |
| chr12 | 51591232 | 52312864 | SCN8A_NM_001330260.2_ENST00000627620.5 | - |
| chr13 | 47733205 | 48270357 | ITM2B_NM_021999.5_ENST00000647800.2 | + |
| chr13 | 48233205 | 48770357 | ITM2B_NM_021999.5_ENST00000647800.2 | - |
| chr13 | 109648962 | 110307157 | COL4A1_NM_001845.6_ENST00000375820.10 | + |
| chr13 | 110148962 | 110807157 | COL4A1_NM_001845.6_ENST00000375820.10 | - |
| chr13 | 109807283 | 110513209 | COL4A2_NM_001846.4_ENST00000360467.7 | + |
| chr13 | 110307283 | 111013209 | COL4A2_NM_001846.4_ENST00000360467.7 | - |
| chr14 | 51504808 | 52069059 | NID2_NM_007361.4_ENST00000216286.10 | + |
| chr14 | 52004808 | 52569059 | NID2_NM_007361.4_ENST00000216286.10 | - |
| chr14 | 72636506 | 73223691 | PSEN1_NM_000021.4_ENST00000324501.10 | + |
| chr14 | 73136506 | 73723691 | PSEN1_NM_000021.4_ENST00000324501.10 | - |
| chr15 | 30501064 | 31101725 | TRPM1_NM_001252024.2_ENST00000256552.11 | + |
| chr15 | 31001064 | 31601725 | TRPM1_NM_001252024.2_ENST00000256552.11 | - |
| chr15 | 50057157 | 50686797 | TRPM7_NM_017672.6_ENST00000646667.1 | + |
| chr15 | 50557157 | 51186797 | TRPM7_NM_017672.6_ENST00000646667.1 | - |
| chr16 | 653105 | 1221768 | CACNA1H_NM_021098.3_ENST00000348261.11 | + |
| chr16 | 1153105 | 1721768 | CACNA1H_NM_021098.3_ENST00000348261.11 | - |
| chr16 | 15203134 | 15857028 | MYH11_NM_001040113.2_ENST00000452625.7 | + |
| chr16 | 15703134 | 16357028 | MYH11_NM_001040113.2_ENST00000452625.7 | - |
| chr16 | 15649564 | 16223494 | ABCC6_NM_001171.6_ENST00000205557.12 | + |
| chr16 | 16149564 | 16723494 | ABCC6_NM_001171.6_ENST00000205557.12 | - |
| chr16 | 29312192 | 29815881 | PRRT2_NM_145239.3_ENST00000358758.12 | + |
| chr16 | 29812192 | 30315881 | PRRT2_NM_145239.3_ENST00000358758.12 | - |
| chr17 | 7810240 | 8322511 | ARHGEF15_NM_173728.4_ENST00000361926.8 | + |
| chr17 | 8310240 | 8822511 | ARHGEF15_NM_173728.4_ENST00000361926.8 | - |
| chr17 | 50060714 | 50627474 | CACNA1G_NM_018896.5_ENST00000359106.10 | + |
| chr17 | 50560714 | 51127474 | CACNA1G_NM_018896.5_ENST00000359106.10 | - |
| chr17 | 63438553 | 63972918 | SCN4A_NM_000334.4_ENST00000435607.3 | + |
| chr17 | 63938553 | 64472918 | SCN4A_NM_000334.4_ENST00000435607.3 | - |
| chr18 | 6441741 | 7117797 | LAMA1_NM_005559.4_ENST00000389658.4 | + |
| chr18 | 6941741 | 7617797 | LAMA1_NM_005559.4_ENST00000389658.4 | - |
| chr18 | 31091876 | 31598821 | TTR_NM_000371.4_ENST00000237014.8 | + |
| chr18 | 31591876 | 32098821 | TTR_NM_000371.4_ENST00000237014.8 | - |
| chr19 | 12706441 | 13506479 | CACNA1A_NM_001127221.2_ENST00000638009.2 | + |
| chr19 | 13206441 | 14006479 | CACNA1A_NM_001127221.2_ENST00000638009.2 | - |
| chr19 | 14659037 | 15200995 | NOTCH3_NM_000435.3_ENST00000263388.7 | + |
| chr19 | 15159037 | 15700995 | NOTCH3_NM_000435.3_ENST00000263388.7 | - |
| chr19 | 17055648 | 17583157 | COLGALT1_NM_024656.4_ENST00000252599.9 | + |
| chr19 | 17555648 | 18083157 | COLGALT1_NM_024656.4_ENST00000252599.9 | - |
| chr19 | 41466581 | 41994230 | ATP1A3_NM_152296.5_ENST00000648268.1 | + |
| chr19 | 41966581 | 42494230 | ATP1A3_NM_152296.5_ENST00000648268.1 | - |
| chr19 | 48657791 | 49211836 | TRPM4_NM_017636.4_ENST00000252826.10 | + |
| chr19 | 49157791 | 49711836 | TRPM4_NM_017636.4_ENST00000252826.10 | - |
| chr20 | 4186455 | 4701588 | PRNP_NM_000311.5_ENST00000379440.9 | + |
| chr20 | 4686455 | 5201588 | PRNP_NM_000311.5_ENST00000379440.9 | - |
| chr20 | 23133656 | 23637955 | CST3_NM_000099.4_ENST00000376925.8 | + |
| chr20 | 23633656 | 24137955 | CST3_NM_000099.4_ENST00000376925.8 | - |
| chr20 | 45508907 | 46016561 | MMP9_NM_004994.3_ENST00000372330.3 | + |
| chr20 | 46008907 | 46516561 | MMP9_NM_004994.3_ENST00000372330.3 | - |
| chr21 | 25380549 | 26170770 | APP_NM_000484.4_ENST00000346798.8 | + |
| chr21 | 25880549 | 26670770 | APP_NM_000484.4_ENST00000346798.8 | - |
| chr21 | 43853620 | 44442644 | TRPM2_NM_003307.4_ENST00000397928.6 | + |
| chr21 | 44353620 | 44942644 | TRPM2_NM_003307.4_ENST00000397928.6 | - |
| chr22 | 16678789 | 17219435 | ADA2_NM_001282225.2_ENST00000399837.8 | + |
| chr22 | 17178789 | 17719435 | ADA2_NM_001282225.2_ENST00000399837.8 | - |
| chrX | 48705062 | 49233340 | CACNA1F_NM_001256789.3_ENST00000323022.10 | + |
| chrX | 49205062 | 49733340 | CACNA1F_NM_001256789.3_ENST00000323022.10 | - |
| chrX | 100897802 | 101407925 | GLA_NM_000169.3_ENST00000218516.4 | + |
| chrX | 101397802 | 101907925 | GLA_NM_000169.3_ENST00000218516.4 | - |
| chrX | 103276839 | 103792619 | PLP1_NM_000533.5_ENST00000621218.5 | + |
| chrX | 103776839 | 104292619 | PLP1_NM_000533.5_ENST00000621218.5 | - |
